# Endothelial-specific deletion of connexin 43 improves renal function and structure after acute kidney injury

**DOI:** 10.1186/s10020-024-01011-6

**Published:** 2024-12-20

**Authors:** Magali Genest, Satoshi Kinugasa, Elena Roger, Louis Boutin, Sandrine Placier, Stefanny Figueroa, Aude Dorison, Safia Hadjadj, Ines Baba, Emmanuel L. Gautier, Panagiotis Kavvadas, Christos Chatziantoniou, Christos E. Chadjichristos

**Affiliations:** 1 Batiment Recherche, INSERM UMR S1155, Tenon Hospital, 4 rue de la Chine, 75020 Paris, France; 2 Cardiovascular Markers in Stress Condition, INSERM, UMR-942, MASCOT, University Paris Cité, 75010 Paris, France; 3 Faculty of Medicine, Sorbonne University, 75013 Paris, France; 4 Critical Care Medicine and Burn Unit, AP-HP, Department of Anesthesiology, FHU PROMICE AP-HP, Saint Louis and DMU Parabol, University Paris Cité, 75010 Paris, France; 5 INSERM, UMR S1166, Sorbonne University, Pitié-Salpétrière Hospital, Paris, France

**Keywords:** Connexin 43, Inflammation, Acute kidney injury

## Abstract

**Background:**

We have previously reported that the gap junction protein connexin 43 (Cx43) was upregulated in chronic renal disease in humans and rodents and plays a crucial role in the progression of experimental nephropathy. In this study, we investigated its role after renal ischemia/reperfusion (rIR), which is a major mechanism of injury in acute renal injury (AKI) and renal transplant graft dysfunction.

**Methods:**

Wild-type mice (WT) and mice in which Cx43 expression was genetically reduced by half (Cx43 ±) were unilaterally nephrectomized. The left renal artery was subsequently clamped, with reperfusion of varying duration. Mice with tubular- or endothelial-specific deletion of Cx43 were also used to assess the effect of this connexin in each cell type after rIR. Kidneys were assessed for histological evaluation, immunohistochemistry, and RT-PCR.

**Results:**

Blood urea nitrogen and creatininemia were progressively elevated in WT mice and picked up 48 h after rIR. At the same time point, severe tubular necrosis and dilation occurred in the cortico-medullary junction of the injured kidneys with accompanying massive neutrophil infiltration. Interestingly, Cx43 expression was progressively increased within the tubulointerstitial compartment during kidney damage progression and was paralleled closely by that of markers of renal dysfunction. Cx43 ± mice showed fewer tubular lesions, less inflammation, and further improved renal function. Similar results were observed in mice where Cx43 was specifically deleted within the vascular endothelium. In contrast, Cx43 deletion in renal tubules did not significantly improve renal structure and function after rIR.

**Conclusion:**

Our findings suggest that endothelial Cx43 plays a crucial role in AKI.

## Introduction

Acute kidney injury (AKI) has been recently associated with an increased risk of mortality and distal organ deleterious effects (Sawhney et al. 2017; Prud’homme et al. 2019; Legrand et al. 2020). Renal ischemia/reperfusion (rIR) is among the major causes of AKI during renal hypoperfusion and transplantation in clinical situations (Legrand et al. 2020). AKI can be promoted by various events including endothelial and epithelial cell injury, inflammation, and extracellular matrix remodelling, followed by tissue repair and re-establishment of renal function. Furthermore, a maladaptive response to AKI has been linked to secondary chronic kidney disease (CKD) and renal fibrosis development (Guzzi et al. 2019).

CKD progression has been recently related to disruptions of gap junction-mediated intercellular communication (GJIC) (Roger et al. 2022). Gap junctions are intercellular channels formed by connexins (Cx), allowing the direct cell-to-cell communication between adjacent cells, via the exchange of ions, small metabolites, and other second messenger molecules (Nielsen et al. 2012). A full gap junction results from the docking of two hemichannels or connexons, assembled from 6 Cx proteins. Hemichannels themselves may play crucial roles in several pathophysiological conditions (Goodenough et al. 2003).

We have recently reported that the expression of Cx43 was highly increased in humans and rodents in renal diseases (Prakoura et al. 2018). This overexpression was deleterious since genetic and pharmacogenetic inhibition of Cx43, improved renal structure and function by inhibiting inflammation and further interstitial renal fibrosis in different models of experimental CKD (Abed et al. 2014; Kavvadas et al. 2017). Some studies also reported a role of Cx43 after IR in tissue injury mainly in the heart and the brain (Gadicherla et al. 2017; Yang et al. 2019). However, the implication of this Cx in renal tissue following acute injury after rIR remains under-investigated.

In this study, we explored the role of Cx43 in AKI after rIR by using mice in which the expression of Cx43 has been genetically reduced by half (Cx43 ±), or specifically deleted in renal tubules or vascular endothelium, as we observed increased expression of Cx43 after AKI in these two cell types. We demonstrated that increased Cx43 expression, from the early stages of the disease, was associated with tubular damage, altered renal function, and neutrophil infiltration. Cx43 ± and mice with specific deletion of Cx43 in endothelial cells, showed limited neutrophil infiltration and improved renal structure and function. In contrast, Cx43-specific deletion in tubular cells didn’t protect against AKI. Thus, endothelial Cx43 seems to play a crucial role in the progression of renal injury after kidney IR.

## Materials and methods

### Animals

Experiments were performed in 2-month-old SV129 males WT and Cx43 ± mice. AKI was induced as previously reported **(**Prud’homme et al. 2019). Briefly, a right nephrectomy and left renal pedicle occlusion (35 min of ischemia) followed by reperfusion were performed under Ketamine/Xylazine anesthesia (intraperitoneal injection of Ketamine: 100 mg/kg and Xylazine: 20 mg/kg). Sham mice underwent the same procedure except for renal pedicle occlusion and right nephrectomy. To get insights into the kinetics of the AKI after renal IR, mice were sacrificed at different time points after reperfusion (3 h, 6 h, 24 h, 48 h, 72 h; n > 7 per each group).

Cx43-tubular specific deletion (Cx43-Tub-Del) in mice was induced as previously described (Roger et al. 2023). Cx43-endothelial specific deletion (Cx43-EC-Del) has been induced by using the same approach as previously detailed (Abed et al. 2021). Only 2-month-old males were used for the rIR experiments and sacrificed after 24 h of reperfusion.

All mice were handled in strict accordance with good animal practice as defined by the relevant national animal welfare bodies of France, and all animal work was approved by the appropriate committee of the National Institute for Health and Medical Research (Inserm) and the Sorbonne University (Paris, France).

### Creatininemia and plasma BUN levels

Blood urea nitrogen (BUN) and creatinine in the blood plasma were obtained on the day of sacrifice, using an enzymatic spectrophotometric method (Konelab automater), and expressed in mmol/L and µmol/L respectively.

### Total RNA extraction, quantitative real time PCR and WB

Total RNA was extracted from half kidneys using TRIzol reagent (Invitrogen). RNA quality was checked by control of optical density at 260 and 280 nm. Contaminating genomic DNA was removed by RNase-free DNAse (Qiagen) for 15 min at room temperature. cDNA was synthesized from 1 µg of purified RNA using oligo-dT and superscript II RT (Qiagen) for 1 h 30 at 37 °C and 10 min at 70 °C. Real-time PCR amplification was performed with Roche Light Cycler 480 Sequence Detection System using SYBR Green PCR Master Mix (Qiagen) as described previously (Abed et al. 2014). All samples were assayed in triplicate, and the average value of the triplicate was used for quantification. Results are expressed as the ratio of a given gene/gene reference (RPL32) cDNA. In the figures, this ratio is mentioned as arbitrary units (AU). The primers used for the study are reported in Table 1.

**Table 1:** Primers used for the study

Gene	Forward primer	Reverse primer
CD68	TTCTGCTGGGAAATGCAAG	AGAGGGGCTGGTAGGTTGAT
CD146	CTGCGAGGCAGAAAGTAACC	ACCCACACCTTCCTCTCCTT
Cx43	GTGCCGGCTTCACTTTC	GGAGTAGGCTTGGACCTTGTC
E-selectin	CAAATCCCAGTCTGCAAAGC	ACATTTCATGTTGCCCTGCT
P-selectin	TCTGGCAGTGTGGACTGG	TCTGGCAGTGTGGACTGG
Gal-3	GTGAAACCCAACGCAAACAG	CCAGTTATTGTCCTGCTTCGT
IL-6	GCTACCAAACTGGATATAATCAGGA	CCAGGTAGCTATGGTACTCCAGAA
IL-10	ACTGCACCCACTTCCCAGT	TGTCCAGCTGGTCCTTTGTT
MCP-1	AGGTCCCTGTCATGTTCTG	TCTGGACCCATTCCTTCTTG
NGAL	CTGAATGGGTGGTGAGTGTGG	CTTGGTATGGTGGCTGGTGG
KIM-1	TCCACTCCTGTCTTTATGCTCC	GTCCCAACCTCTATCACACCTG
RPL32	GCTGCCATCTGTTTTACGG	TGACTGGTGCCTGATGAACT

For Western blotting, proteins from half kidneys were extracted as previously reported (Abed et al. 2021). Immunoblotting was performed for Cx43 (Abcam #11370, 1/2000) and Gal3 (Abcam #ab53082, 1/1000). GAPDH (Sigma #G9545, 1/100000) was used as a loading control.

### Renal morphometry and immunohistochemistry

Three µm thick cryostat sections were placed onto super Frost^®^glass slides (Menzel GmbH & Co KG). Immunostainings for Cx43 (Sigma-Aldrich C6219, 1/200), and CD146 (R&D Systems AF6106-SP, 1/200) were performed on acetone-fixed sections as previously described (Abed et al. 2014; Kavvadas et al. 2017). For neutrophil detection, 4 µm thick paraffin-embedded sections were immunostained with anti-Gr1 (Ly-6C/Ly-6G) antibody (BD Pharmigen #55029, 1/100) as previously described (Kormann et al. 2020).

For tubular lesions, paraffin-embedded sections were stained with Periodic Acid-Schiff (PAS). Tubular dilation and necrosis were evaluated semi-quantitatively in PAS-stained slides using the following scale: 0, no tubular damage; 1, damage in 1–20% of the tubules analyzed; 2, damage in 26–40% of the tubules analyzed; 3, damage in 41–60% of the tubules analyzed; 4, damage in > 61% of the tubules analyzed, and the mean value was calculated for each mouse. Tubular injury was evaluated in blind, as simultaneous quantification of both tubular dilation and necrosis. All samples were analyzed an Olympus BX50 inverted microscope. Immunostainings have been confirmed using at least 5 mice from each group. Negative controls included omission of first antibodies or preincubation of first antibodies with immunogenic peptides.

### Statistical analyses

Values are expressed as mean ± SEM. Data were analyzed using one-way analysis of variance followed by protected least significant difference Fisher’s test of the Statview software package. Results with P < 0.05 were considered statistically significant.

## Results

### Decreased expression of Cx43 improved kidney structure and function after renal IR

**Fig. 1:**
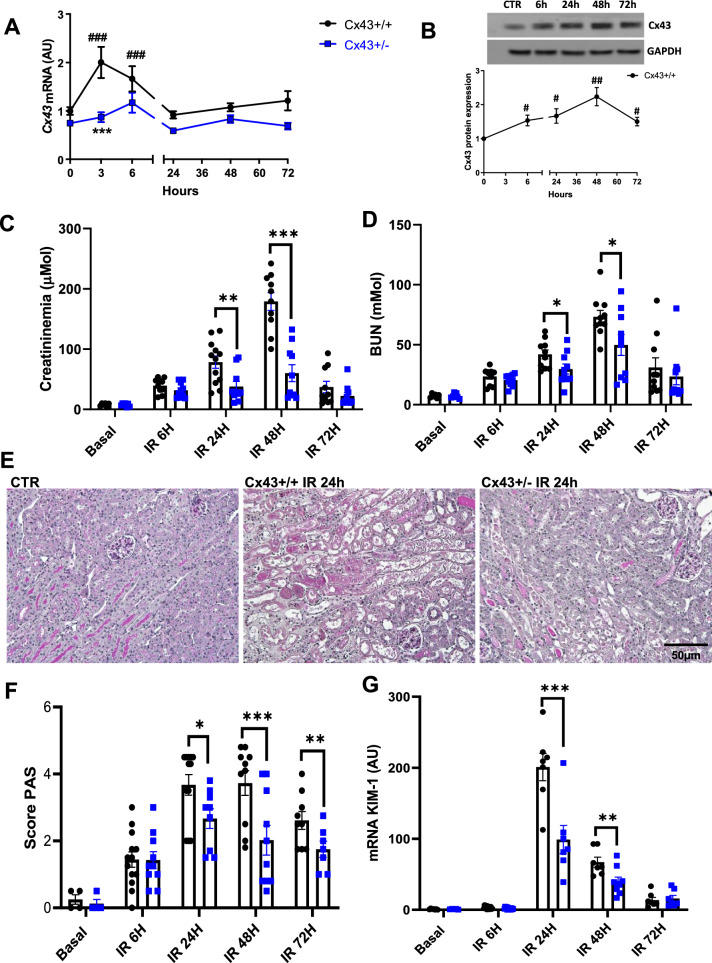
Cx43 ± mice showed improved renal function and structure after IR. Cx43 expression is progressively increased at the mRNA (**A**) and protein levels (**B**) in injured kidneys after renal IR. Renal function was evaluated by plasma creatinine (**C**) and BUN (**D**) measurements. All parameters reveal a slower progress of the disease in Cx43 ± animals. Representative images of PAS staining (**E**) show that renal structure was preserved in Cx43 ± vs Cx43 + / + since they developed less tubular necrosis and dilation at 24, 48 and 72 h after IR (**F**). qPCR for KIM-1 confirmed tubular protection in Cx43 ± mice (**G**). Values are presented as mean ± SEM; n = 7–10 mice from each group; *P < 0.05, **P < 0.01, ***P < 0.001. * compares Cx43 + / + versus Cx43 ± , # fold induction initial versus different time points. Scale bar, 50 μm

To first study the alteration of Cx43 expression in the kidney after IR renal disease, WT mice were sacrificed at different time points after reperfusion. Quantitative RT-PCR analysis showed that the Cx43 mRNA expression was significantly increased at the early stages of the disease, after 3 h of IR (Fig. 1A) followed by an increase at the protein level at 6 h that picked up 48 h after IR (Fig. 1B). Increased Cx43 expression was paralleled closely by that of clinical markers of renal dysfunction. Indeed, after renal IR in WT mice, a transient increase in plasma creatinine and blood urea nitrogen (BUN) levels were observed at 24 and 48 h post-reperfusion (Fig. 1C, D). To further investigate the role of Cx43 in AKI we used Cx43 ± mice. Of note, it was not possible to use Cx43–/– as they die shortly after birth (Reaume et al. 1995). As expected, induction of Cx43 expression following rIR was blunted in Cx43 ± mice after IR. Furthermore, in these mice, renal function was substantially improved as the increase of both creatininemia (78.3 ± 10 µM for Cx43 + / + versus 37.9 ± 8 µM for Cx43 ± after 24 h, and 179 ± 14 µM for Cx43 + / + versus 60.1 ± 13 µM for Cx43 ± after 48 h) and BUN was less pronounced (42 ± 3.6mMol for Cx43 + / + versus 29.7 ± 4 mM after 24 h, and 73.3 ± 5.1 mM for Cx43 + / + versus 49.8 ± 8.3 mM for Cx43 ± after 48 h). In accordance, structural damages such as tubular necrosis and dilation within the cortico-medullary junction were significantly improved in Cx43 ± mice from 24 h after rIR (3.65 ± 0.3 for Cx43 + / + versus 2.7 ± 0.3 for Cx43 ± after 24 h of rIR, 3.73 ± 0.3 for Cx43 + / + versus 2.02 ± 0.3 for Cx43 ± after 48 h of rIR, and 2.6 ± 0.25 for Cx43 + / + versus 1.75 ± 0.22 for Cx43 ± after 72 h of rIR) (Fig. 1E, F). This protection was confirmed by measurements of KIM-1 mRNA, a well-established marker of tubular injury (Fig. 1G). These data show that reduced expression of Cx43 improves both renal structure and function during AKI.

### Inflammation was restricted in Cx43 ± mice after IR

**Fig. 2:**
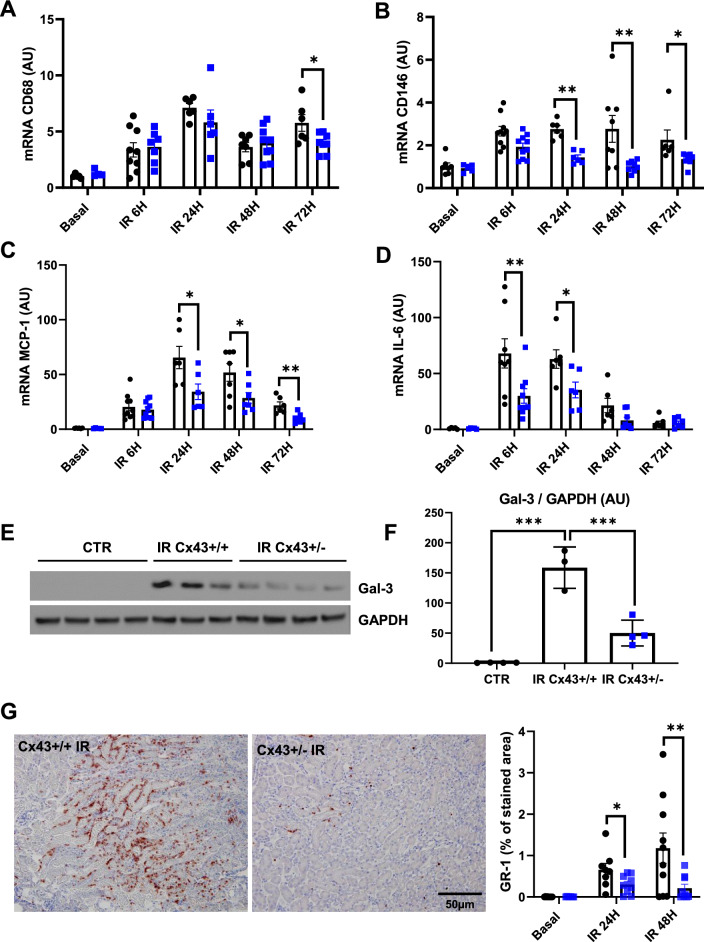
Cx43 ± mice develop less renal inflammation after AKI. qPCR for CD68 (**A**), CD146 (**B**), MCP-1 (**C**) and IL-6 (**D**) show that increased expression of mRNAs of all these inflammatory markers was blunted in Cx43 ± kidneys at distinct time points after AKI. Representative western blots for Gal-3 and GAPDH were performed by using renal cortex of sham controls and kidneys after 24 h of IR (**E**). Graphs show quantification of western blots expressed as the ratio of Gal3 versus GAPDH signal for each sample (**F**). Gal-3 increased expression was moderated in Cx43 ± mice 48 h after renal IR. At the same time point, quantification of immunohistochemistry for GR1 indicate that Cx43 ± develop less inflammation compared to Cx43 + / + mice (**G**). Values are presented as mean ± SEM; n = 7–10 mice from each group except for WB (n = 3–4 mice per group); *P < 0.05, **P < 0.01, ***P < 0.001. ● represents Cx43 + / + , and ■ Cx43 ± . Scale bar, 50 μm

We have previously reported that Cx43 increased expression was related to inflammation in renal diseases (Prakoura et al. 2018). Consequently, we first evaluated the expression of some inflammatory markers by qPCR. As expected, mRNA expressions of the monocyte marker CD68, the endothelial dysfunction marker CD146, the monocyte chemoattractant protein-1 (MCP-1), and the interleukin-6 (IL-6), were significantly upregulated since 6 h of renal IR. At distinct time points after renal IR, this upregulation was reduced for nearly all the above-mentioned markers in Cx43 ± mice (Fig. 2A–D). Similar results were observed regarding the protein expression of Galectin-3 (Gal-3), a well-known lectin that is highly involved in inflammatory cell adhesion during kidney damage (Prud’homme et al. 2019; Boutin et al. 2022). Indeed, western blotting for Gal-3 showed that this proinflammatory protein was induced in WT animals after 48 h of rIR, and its induction was blunted in Cx43 ± mice (Fig. 2E, F). In addition, GR1 immunostaining showed very limited neutrophil infiltration in the cortico-medullary junction in these mice after 24 and 48 h of rIR (Fig. 2G). Thus, decreased Cx43 expression markedly restricted renal inflammation after AKI.

### Cx43 is overexpressed in endothelial and tubular damaged renal cells

**Fig. 3:**
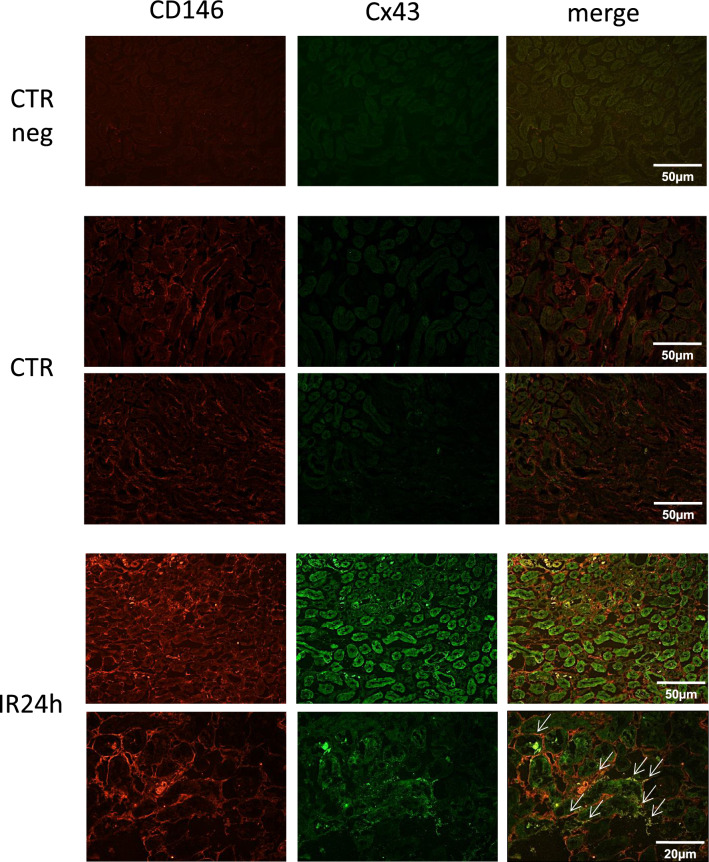
Cx43 expression was mainly increased within damaged tubules and renal peritubular capillaries after IR. Immunofluorescence showed increased expression of Cx43 in damaged tubules after 24 h of renal IR. Double immunofluorescense for Cx43 (green) and the endothelial marker CD146 (red) in damaged kidneys indicate localization of Cx43 in renal microcirculation (white arrows) after 24 h of renal IR. Scale bars 50 μm and 20 μm. Negative controls (CTR neg) included the omission of primary antibodies

To identify the renal cell type in which Cx43 was increased we performed immunostainings using cryosections sections of kidneys before and after renal IR (Fig. 3). At basal conditions Cx43 was almost indetectable within renal tissue, as we have reported in previous studies (Toubas et al. 2011; Abed et al. 2014). In the same conditions, the endothelial marker CD146 was slightly expressed in renal endothelial cells (Abed et al. 2021). After 24 h of rIR Cx43 expression was induced within renal tubules. At the same time point, the increased expression of CD146 was found to be colocalized with that of Cx43, particularly in the peritubular capillaries. Thus, Cx43 is abnormally expressed in both endothelial and renal tubular cells after aggression during the progression of AKI.

### Cx43 tubule-specific deletion does not improve renal damage after AKI

**Fig. 4:**
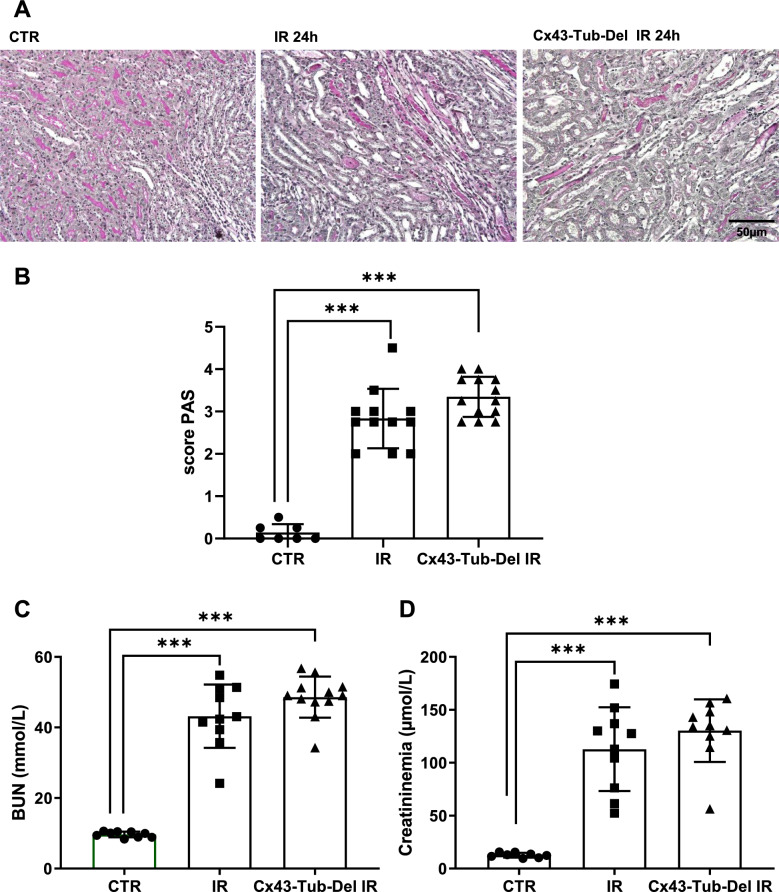
Cx43-specific deletion in renal tubules does not affect renal structure and function after AKI. Representative images of PAS staining (**A**) show that renal structure was not preserved in Cx43-Tub-Del mice (**A**) since tubular damage was similar in mice with and without tubule-specific deletion of Cx43 after 24 h of renal IR (**B**). Both BUN (**C**) and creatininemia measurements showed similar data regarding renal function (**D**). Values are presented as mean ± SEM; n = 7–12 mice from each group; *P < 0.05, **P < 0.01, ***P < 0.001. Scale bar 50 μm

**Fig. 5:**
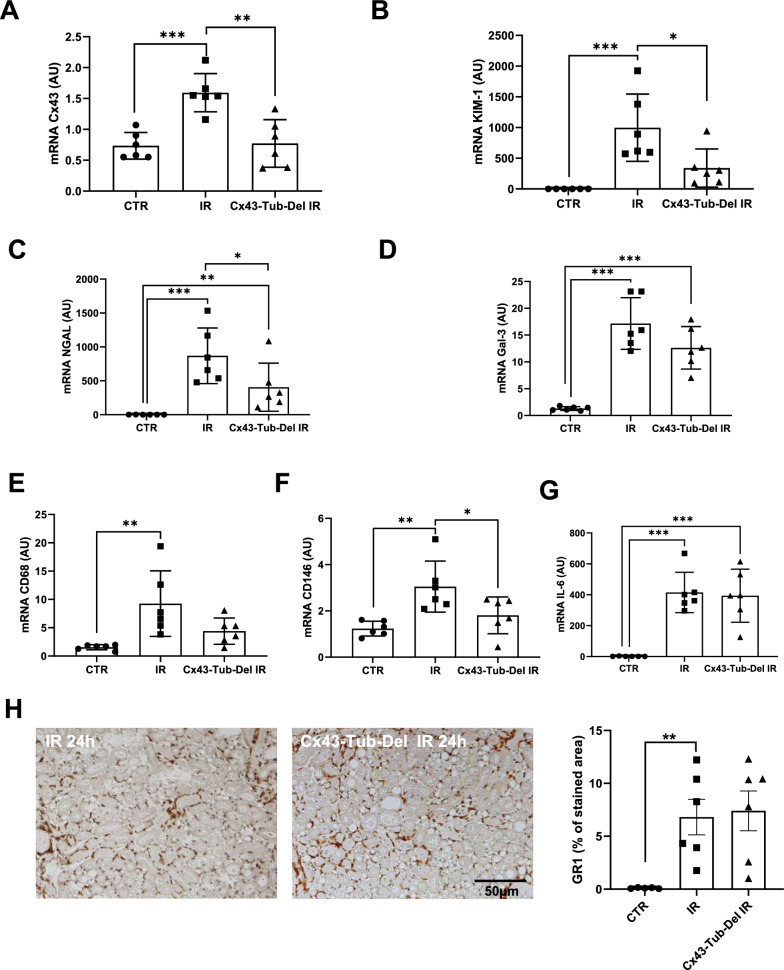
mRNA expression for tubular and inflammatory markers in kidneys after renal IR. qPCR for Cx43 (**A**), KIM-1 (**B**), NGAL (**C**), Gal3 (**D**), CD68 (**E**), CD146 (**F**) and IL-6 (**G**), using kidneys from mice with and without Cx43 tubular-specific deletion after 24 h of renal IR. Data are expressed in graphs as arbitrary units (AU) that represents the ratio of the target gene/internal control gene (RPL32). Representative immunostainings for GR1 and its quantification (**H**). n = 6 mice per group. *, P < 0.05; **, P < 0.01; ***, P < 0.001

To assess the role of the Cx43 increased expression in damaged tubules, we created the Cx43-Tub-Del mouse strain, in which deletion of this Cx was specifically induced in renal tubular cells (Roger et al. 2023). Cx43-Tub-Del mice showed no significant differences in tubular damage (2.8 ± 0.67 for IR versus 3.3 ± 0.45 for Cx43-Tub-Del mice), BUN (43.2 ± 8.5 mM for IR versus 48.5 ± 5.6 mM for Cx43-Tub-Del mice) and plasma creatinine (112.8 ± 39 µM for IR versus 130 ± 29.6 µM for Cx43-Tub-Del mice) after 24 h of renal IR, compared to mice in which Cx43 was not specifically deleted in tubular cells (indicated as IR group) (Fig. 4A–D). However, Cx43 increased expression after 24 h of IR was blunted in Cx43-Tub-Del mice (Fig. 5A). In addition, mRNA increased expression for some tubular markers such as KIM-1 and N-GAL, and proinflammatory proteins such as CD146 were also blunted in Cx43-Tub-Del mice. However, Gal-3, CD68 and IL-6 mRNA levels were similar between Cx43-Tub-Del and IR groups (Fig. 5B–G). In addition, GR1 immunostaining showed no differences in neutrophil infiltration, in renal tissues of mice with and without Cx43 tubular-specific deletion after 24 of rIR (Fig. 5H). In conclusion, although mRNA levels for some tubular and proinflammatory markers seem to have improved, histological tubular damage and renal function were similar whether Cx43 was deleted or not in renal tubules.

### Cx43 endothelial-specific deletion improves renal structure and function, and limits tissue inflammation after AKI

**Fig. 6:**
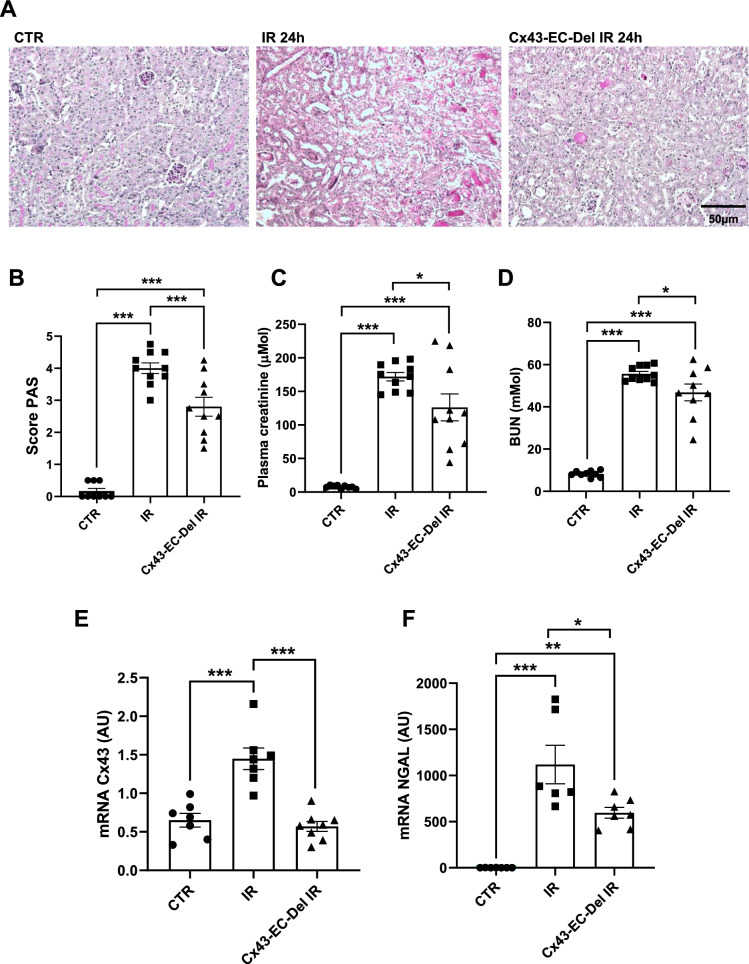
Cx43-specific deletion within vascular endothelium improves renal structure and function after AKI. Representative images of PAS staining (**A**) show that kidney structure was preserved in Cx43-EC-Del mice, since we observed less tubular necrosis and dilation after 24 h of renal IR (**B**). Renal function was evaluated by creatininemia (**C**) and BUN (**D**) measurements. qPCR for Cx43 (**E**) and NGAL (**F**) are presented in graphs as arbitrary units (AU), that represent the ratio of the target gene/internal control gene (RPL32). Values are presented as mean ± SEM; n = 7–10 mice from each group; * *P < 0.05, **P < 0.01, ***P < 0.001. Scale bar, 50 μm

**Fig. 7:**
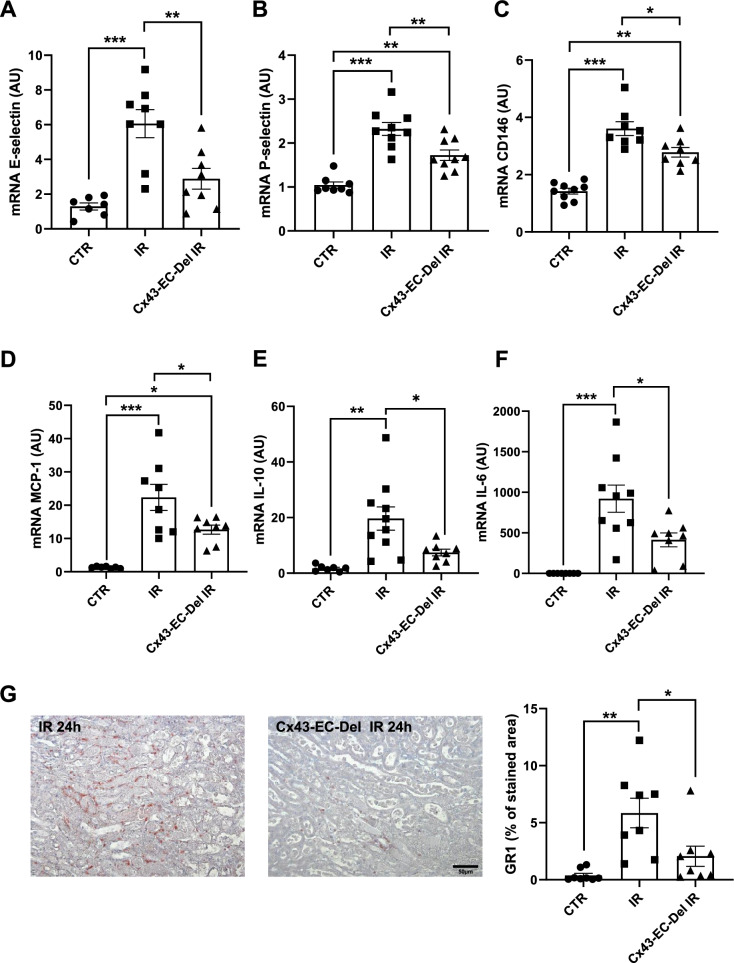
Cx43-specific deletion within vascular endothelium protects against renal inflammation after AKI. mRNA expressions for E-selectin (**A**), P-selectin (**B**), CD146 (**C**), MCP-1 (**D**), IL-10 (**E**) and IL-6 (**F**), in kidneys from mice with and without Cx43 endothelial-specific deletion after 24 h of renal IR. At the same time point, quantification of GR1 immunostaining (**G**) indicates that Cx43-CE-Del developed less inflammation compared to mice in which Cx43 was overexpressed. qPCR data are illustrated in graphs as arbitrary units (AU), representing the ratio of the target gene/internal control gene (RPL32). Values are presented as mean ± SEM; n = 7–10 mice from each group; * *P < 0.05, **P < 0.01, ***P < 0.001. Scale bar, 50 μm

To study the role of the Cx43 increased expression within renal vascular endothelium, we created the Cx43-EC-Del mouse strain, in which deletion of this Cx was specifically induced in endothelial cells (Abed et al. 2021). Cx43 endothelial-specific deletion protected from severe tubular damage after 24 h of renal IR (4 ± 0.16 for IR versus 2.8 ± 0.3 for Cx43-EC-Del mice) (Fig. 6A, B). In accordance, plasma creatinine (171.8 ± 6 µM for IR versus 124.1 ± 19 µM for Cx43-Tub-Del mice) and BUN values (55.6 ± 1 mM for IR versus 46.9 ± 3.7 mM for Cx43-Tub-Del mice) were both decreased in Cx43-EC-Del mice at this time point, showing preserved renal function (Fig. 6C, D). Furthermore, in these mice, the decrease in the expression of renal Cx43 mRNA was similar to that of NGAL (Fig. 6E, F). In addition, the endothelial activation markers E- and P-selectin and CD146 were attenuated after 24 h of renal IR, while the expression of proinflammatory cytokines such as MCP-1, IL-10, and IL-6 was decreased in Cx43-EC-Del mice (Fig. 7A–F). Finally, the above observations were associated with lower inflammatory cell infiltration, assessed by immunostainings for the neutrophil marker GR-1 (Fig. 7G). Thus, endothelial-specific deletion of Cx43 within the vascular endothelium protected mice after AKI.

## Discussion

In this study, we investigated the role of Cx43 in rIR-induced AKI. We demonstrated that the expression of this Cx was progressively increased during the progression of the disease. Such an overexpression was deleterious since Cx43 ± mice showed improved renal function and structure after IR. Similar results were observed in mice where Cx43 was specifically deleted within the renal vascular endothelium. Indeed, our study has demonstrated that the upregulation of Cx43 was colocalized with the endothelial marker CD146, which is known to be highly upregulated in the injured endothelium of the kidney during the progression of renal disease (Abed et al. 2021). CD146 upregulation was associated with the transmigration of inflammatory cells across the damaged endothelium in vascular diseases, reinforcing the hypothesis that increased expression of this Cx in the injured endothelial cells may play a crucial role in the early stages of AKI (Bardin et al. 2006, 2009; Boutin et al. 2024). It’s interesting also to note that there are disparate differences in injuries after 24 h of rIR, among experiments conducted on control mice. This is probably due to the heterogenicity of our experimental model of renal IR, especially when the experiments were completed in lengthy lap periods and by different operators. However, the fact that renal injury is more severe in the Cx43 endothelial-specific deletion experiments when compared to control mice, reinforces our findings about the harmful impact of the Cx43 overexpression by endothelial cells on the development of AKI.

AKI is linked with both short and long-term risk of mortality in different situations. Indeed, AKI is associated with an increased risk of cardiac events in humans and rodents (Burchill et al. 2008; Parikh et al. 2017; Gammelager et al. 2014). The consequences of cardiovascular events after AKI have been reported in patients during the year following hospital discharge. Indeed, using a large database Go et al. confirmed that AKI was a risk factor for heart failure during the year following discharge (Go et al. 2018). Furthermore, a maladaptive response to AKI may contribute to the development of chronic kidney disease (Basile et al. 2012). Consequently, a better understanding of the mechanisms governing renal injury and repair may lead to a more efficient management of patients suffering from AKI.

Our data are in accordance with our previous publications in the context of CKD. Indeed, in different models of experimental CKD, targeting distinct renal compartments, the expression of Cx43 was increased or de novo expressed in damaged renal cells (Roger et al. 2022). Thus, in hypertensive mice, Cx43 was highly increased within renal vasculature, following ureteral unilateral obstruction within the tubule-interstitial compartment, and after induction of experimental glomerulonephritis, Cx43 was increased in glomerular endothelial cells and de novo expressed in podocytes (Abed et al. 2014). Cx43 overexpression was deleterious because Cx43 ± mice were protected against the progression of experimental CKD. Moreover, targeting Cx43 with specific antisense reversed in some models the progression of renal disease. Thus, our data confirmed that the protective effect of the Cx43 blockade is not model-dependent and can be considered as a more common protective mechanism of renal disease.

The importance of Cx43 in modulating the severity of inflammatory disease has been already established in several diseases (O’Carroll et al. 2013). In our study, we showed that Cx43 expression was enhanced in damaged kidneys and associated with the induction of inflammatory indexes leading to pronounced neutrophil infiltration in the cortico-medullary junction. Given our previous observations in CKD experimental models, we assume that this Cx may contribute to the recruitment of inflammatory cells at the early stages of the disease. An increased expression of the Cx43 has also been reported during the progression of chronic vascular inflammatory diseases such as atherosclerosis (Kwak et al. 2002). Interestingly, reducing Cx43 expression restricted atherosclerotic plaque development in mice (Kwak 2003). In addition, atherosclerosis-susceptible mice expressing reduced levels of Cx43 displayed restricted intimal thickening, following angioplasty (Chadjichristos et al. 2006). The reduction of Cx43 resulted in decreased inflammatory cell infiltration in the injured site of the vessel and was associated with accelerated vascular repair. Similar to our study, it has been reported that using a Cx43 endothelium-specific deletion in mice, the TNF-α-induced leukocyte adhesion and transmigration were greatly reduced (Véliz et al. 2008). In addition, Cx43 may serve as a conducting pathway by amplifying Ca + 2-signalling between endothelial cells to spread inflammatory signals within the lung capillary network (Parthasarathi et al. 2006). In the same tissue, Cx43 ± mice displayed reduced neutrophil recruitment, after intratracheal instillation of LPS (Sarieddine et al. 2009). Similarly, Mori et al. showed that recruitment of neutrophils was markedly reduced after Cx43 blockade suggesting that by reducing Cx43 expression early in the skin, the wound healing process was enhanced due to inflammatory response attenuation (Mori et al. 2006). Finally, blocking Cx43 upregulation in different models of spinal cord injury or in corneal scrape injury in rodents reduced inflammation and improved functional recovery (Cronin et al. 2008; Nakano et al. 2008). In accordance with our data, all the above-mentioned studies underline the interest for targeting specifically Cx43 to protect against the progression of inflammatory diseases.

Renal IR affects mainly the tubulo-interstitial compartment. The nature of tissue healing is dependent on the degree to which the injured cells can recover and restore normal renal function. Subsequent studies revealed a direct effect of Cx43 in protecting renal tubular cells from damage. We have reported that blocking Cx43 with specific antisense attenuated E-cadherin down-regulation, a marker of renal tubular epithelial phenotype in obstructive nephropathy (Abed et al. 2014). In the same experimental model, Cx43 ± mice showed less cell death caused by a number of stressors resulting from ureteral obstruction including ischemia, hypoxia or axial strain caused by tubular dilatation. The use of Cx43-specific blocking peptides in vitro preserved renal tubular phenotype upon TGF-β1 stimulation. These data are in accordance with our study reporting limited tubular damage in Cx43 ± mice. A recent study, in the context of diabetic nephropathy, suggested that chronic exposure to glucose-evoked TGFβ1 induced an increase in Cx43 expression, consistent with changes observed in renal tubular epithelia from patients. Despite increased Cx43 expression, direct gap junction intercellular communication decreased, whilst hemichannel expression/function and paracrine release of ATP increased, leading to increased expression of interleukin 6 and fibronectin. The authors demonstrated that exacerbated purinergic signaling may support the progression of tubular damage (Hills et al. 2018). We have also recently reported that aberrant Cx43-hemichannel activity in renal tubular cells contributed to tubule inflammation via activation of the NLRP3 inflammasome. Interestingly, the use of a specific Cx43-hemichannel blocker conferred protection against tubulointerstitial injury using the unilateral ureteral obstruction model of chronic kidney disease (Roger et al. 2023). Furthermore, Cx43-tubule-specific deletion prior to UUO protected against tubular injury. However, in our study, Cx43 deletion in tubular cells didn’t protect against tubulointerstitial injury after AKI, at least 24 h after renal IR. Indeed, tissue damage and altered renal function showed no improvement following this deletion. In addition, immunostainings for infiltrating neutrophils did not reveal any obvious difference between mice with or without Cx tubular-specific deletion after renal IR. However, some beneficial effects were observed for the tubular damage markers KIM-1 and NGAL, but only at the mRNA levels. Thus, a protective effect of the Cx43 tubule-specific deletion cannot be excluded at later time points, but in this study, we focused exclusively on earlier stages of the disease, since structural and functional protection was observed in Cx43 ± mice after 24 h of renal IR.

## Conclusion

Our study clearly demonstrated for the first time a deleterious role of endothelial Cx43 in AKI after renal IR. Although further work is required to clarify molecular insights involving Cx43 in the progression of cell damage and repair after renal IR, our study shows that this Cx may be a potential therapeutic target against AKI.

## Data Availability

All data generated and analyzed during the current study are available from the corresponding authors on reasonable request.
